# Wearable device–measured physical activity and risk of MAFLD in adolescents

**DOI:** 10.1016/j.ajpc.2025.101345

**Published:** 2025-11-03

**Authors:** Ruli Wang, Congwei You, Zijuan Dong, Qingqing Zheng, Xiaowei Zheng, Liangbin Zhou, Xiaolei Wang, Le Zhang, Haoyang Zhang

**Affiliations:** a Department of Paediatrics, Affiliated Children’s Hospital of Jiangnan University (Wuxi Children’s Hospital), Wuxi, China; b Department of Paediatrics, Affiliated Wuxi People's Hospital of Nanjing Medical University, Wuxi, China; c Public Health Research Center and Department of Public Health and Preventive Medicine, Wuxi School of Medicine, Jiangnan University, Wuxi, China; d Department of Biomedical Engineering, Faculty of Engineering, The Chinese University of Hong Kong, Hong Kong, China; e Department of Experimental Medical Science, Lund University, Lund, Sweden

**Keywords:** Physical activity, MAFLD, Wearable devices, Adolescent

## Abstract

**Background:**

While physical activity is known to affect the risk of MAFLD in adults, evidence in adolescents is limited and often based on self-reported questionnaires. We therefore used wearable device–based measurements to investigate the association between physical activity and MAFLD in adolescents.

**Methods:**

Data were obtained from the National Health and Nutrition Examination Survey (NHANES) database for adolescents aged 12–19 years during the 2003–2006 and 2011–2014 cycle. Physical activity was measured using wearable accelerometers, averaged across all valid wearing days. Weighted multivariate logistic regressions were applied to assess associations between physical activity (overall, daytime, and nighttime) and MAFLD.

**Result:**

Among 5705 adolescents, higher physical activity levels were strongly associated with lower odds of MAFLD in the 2003–2006 cycle, with a clear dose–response trend across quartiles (*P* for trend < 0.001). Participants in the highest physical activity (Q4) had 60 % lower odds overall, 58 % lower odds for daytime, and 43 % lower odds for nighttime activity, compared with those with lowest physical activity (Q1). The association between higher physical activity and lower MAFLD risk was generally observed across sex, age, and income subgroups. In contrast, no significant association between physical activity and MAFLD risk was observed in the 2011–2014 cycle.

**Conclusion:**

Our results suggest that higher levels of physical activity, especially during the daytime, may protect against MAFLD in adolescents. Our findings offer partial evidence for the role of circadian activity patterns in MAFLD risk and underscore the need for future studies to validate these associations.

## Graphical abstract

**Figure:**
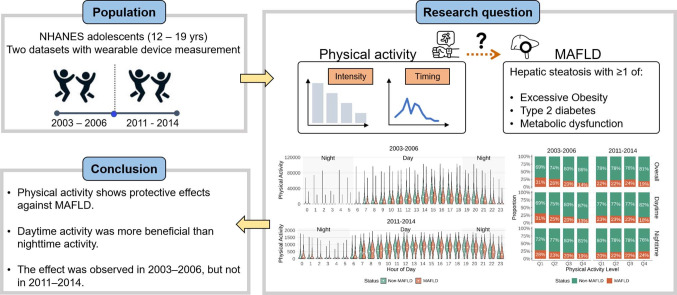

## Introduction

Metabolic Dysfunction-Associated Fatty Liver Disease (MAFLD), formerly referred to as non-alcoholic fatty liver disease (NAFLD), is a chronic liver disease closely associated with metabolic dysfunction and its global incidence is on the rise [1,2]. The global prevalence of pediatric MAFLD is estimated at 7.4 %, reaching 8.53 % in Asia, and continues to rise annually [3]. This growing burden is especially concerning, as studies have indicated that pediatric MAFLD or NAFLD may progress more aggressively than in adults [4,5], with evidence pointing to greater severity of liver fibrosis and disease progression in younger patients [6,7]. While the incidence of chronic liver disease in children is low [8], it tends to progress rapidly when it occurs, often resulting in liver fibrosis and cirrhosis [9]. Pharmacological intervention in children is controversial, and lifestyle changes and adjustments remain the primary treatment for pediatric MAFLD [10]. In most patients, a weight loss of 7 %–10 % is described as reversing MAFLD [10,11].

Adolescence is widely recognized as a transitional period from childhood to adulthood, marked by a second pubertal growth spurt, and is a critical phase for shaping future health and well-being [12]. In recent years, lifestyle patterns across all age groups have shifted markedly, with adolescents particularly affected [13]. Compared with previous generations, contemporary youth engage in lower levels of physical activity and spend more time in sedentary behaviors such as screen-based entertainment [14,15]. Limited physical activity or frequent complete lack of physical activity can result in various health issues, including postural problems [16,17], physical conditions [18], overweight and obesity [19], circulatory problems [20,21], and even premature death [22]. To date, no study has used wearable devices to objectively measure physical activity in pediatric populations and examine its association with MAFLD. In NHANES, interview data for children under 16 years are typically provided by proxy respondents, which can lead to incomplete or imprecise reporting. Additionally, children often engage in sporadic physical activity outside the home, making it difficult for proxies to accurately recall and quantify their behavior. To address these limitations, this study utilizes wearable devices to objectively measure adolescents’ physical activity and examines the association between activity intensity, timing (daytime vs. nighttime), and MAFLD in this population.

## Methods

### Study population

This study utilized data from the NHANES, a nationally representative program designed to assess the health and nutritional status of children and adults in the United States (available on https://wwwn.cdc.gov/nchs/nhanes/). The survey was approved by the Research Ethics Review Committee of the National Center for Health Statistics, and all participants provided informed consent.

**Fig. 1:**
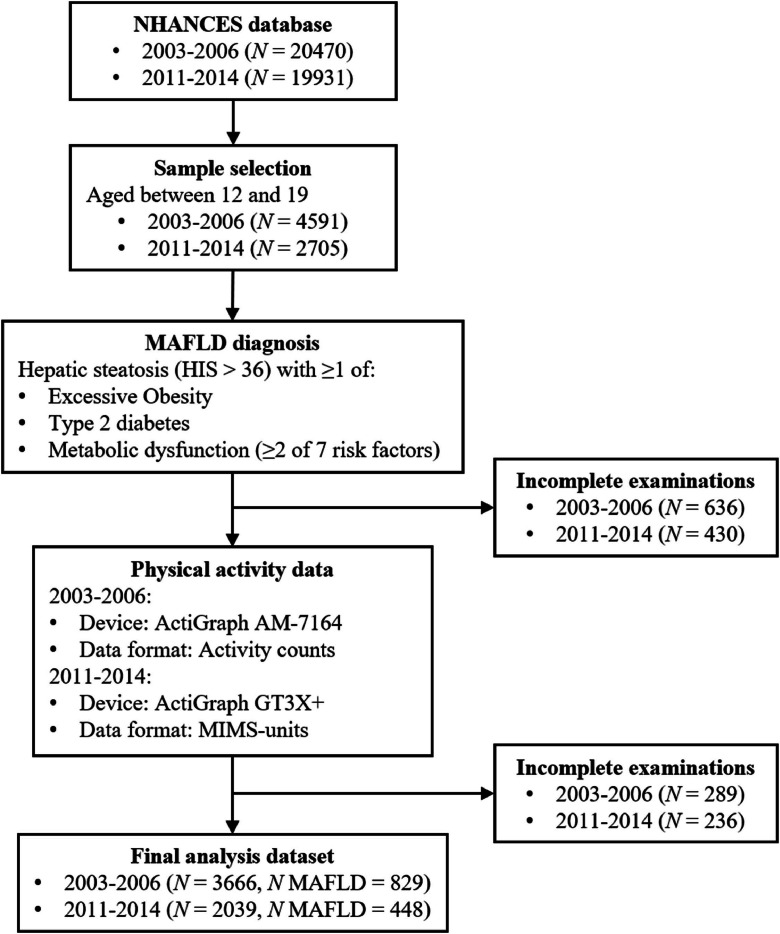
Flow diagram of data collection and participant selection.

We included participants from two NHANES cycles (2003–2006 and 2011–2014), during which wearable accelerometers were used to objectively measure physical activity. The inclusion criteria for the final participant selection were as follows: (1) adolescent aged between 12 and 19 years old; (2) exclusion of those with missing values for MAFLD and physical activity. A total of 3666 participants were included from the 2003–2006 period, and 2039 participants were included from the 2011–2014 period. The flow of participants is illustrated in Fig. 1.

Adolescents aged 12–19 from NHANES 2003–2006 and 2011–2014 were included. Physical activity was measured by accelerometers, and MAFLD was defined based on hepatic steatosis with metabolic risk factors. After exclusions, the final samples were 3666 (829 MAFLD) in 2003–2006 and 2039 (448MAFLD) in 2011–2014.

### Wearable device–measured physical activity

The physical activity monitor was added to the NHANES database in 2003, with the main objective of collecting objective information on physical activity. In the NHANES database from 2003 to 2006, physical activity was monitored using the ActiGraph AM-7164, a device manufactured by ActiGraph. This monitor records vertical accelerations as “counts”, which represent the relative intensity of each movement. Participants were instructed to wear the monitor over their right hip during waking hours and to remove it only for activities such as swimming or bathing. The data were collected in 1-minute epochs over a period of 7 days.

From 2011 to 2014, physical activity was assessed using the ActiGraph GT3X+ accelerometer, which was manufactured by ActiGraph in Pensacola, Florida. Participants were instructed to wear the device on their non-dominant wrist and were advised not to tamper with it, except for wearing it as directed. The device measured acceleration on three axes (x, y, and z) at a sampling rate of 80. For this study, we utilized the summary datasets produced and released by NHANES, which included individual-level data summarized in monitor-independent movement summary units (MIMS-units) by minute, hour, and day.

### Demographic and clinical characteristics

Basic demographic characteristics include age, sex, race (non-Hispanic White, non-Hispanic Black, Mexican American, or other), ratio of family income to poverty (PIR), body mass index (BMI), alanine aminotransferase (ALT), and aspartate aminotransferase (AST), systolic blood pressure (SBP), diastolic blood pressure (DBP), obesity, and diabetes. PIR is used to categorize household income levels into low (PIR<1.3), middle (PIR: 1.3–3.5), and high (PIR>3.5). ALT and AST are included in biochemical analysis. SBP and DBP are the average of three measurements. Excess adiposity is diagnosed using BMI and waist circumference. Adolescent diabetes is determined based on self-reported questionnaire data combined with blood glucose concentration and glycated hemoglobin (HbA1c) levels.

### MAFLD diagnosis

In accordance with the International Expert Consensus on Pediatric MAFLD, the diagnostic criteria for MAFLD are based on hepatic fat accumulation (steatosis), as well as one of the following three criteria: excess adiposity, presence of type 2 diabetes (T2DM), or evidence of metabolic dysfunction [23].

For hepatic fat accumulation, we used the Hepatic Steatosis Index (HSI) [24] to define hepatic steatosis: HSI = ALT/AST + BMI + 2 (if diabetic) + 2 (if female). A score greater than 36 is associated with the presence of hepatic steatosis.

Childhood excess adiposity was classified into overweight and obesity and abdominal obesity. Overweight and obesity were defined according to the WHO Child Growth Standards: for children under 5 years of age, a BMI greater than 2 standard deviations above the mean; and for those aged 5 to 15 years, a BMI greater than 1 standard deviation above the mean. Abdominal obesity was defined as a waist circumference greater than the 90th percentile for age and sex.

Childhood diabetes was defined by any of the following: a self-reported diagnosis by a physician, a fasting blood glucose level equal to or greater than 126 mg/dL, or a HbA1c level of 6.5 % or higher.

Metabolic dysfunction is defined as the presence of at least two or more metabolic risks out of the following seven items, according to gender and age percentiles. For children aged 2 to 9 years, the presence of any two or more of the following metabolic abnormalities qualified as metabolic dysfunction: serum triglyceride concentration above the 90th percentile, serum HDL cholesterol at or below the 10th percentile, systolic or diastolic blood pressure above the 90th percentile, or a triglyceride-to-HDL cholesterol ratio greater than 2.25. For children aged 10 to 15 years, the criteria included systolic blood pressure greater than 130 mmHg or diastolic pressure greater than 85 mmHg, serum triglycerides greater than 1.5 g/L, HDL cholesterol <0.4 g/L, or a triglyceride-to-HDL ratio greater than 2.25. For individuals aged 16 years and older, the same diagnostic criteria used for adults were applied.

### Statistical analysis

Due to differences in the measurement devices used during the two time periods, data from 2003 to 2006 and 2011–2014 were processed separately. For descriptive and comparative analyses, categorical variables were summarized as counts and percentages, while continuous variables were reported as means with standard deviations (SD). Differences between MAFLD and non-MAFLD groups were assessed using the chi-square test for categorical variables and the Wilcoxon rank-sum test for continuous variables.

To assess the relationship between physical activity and MAFLD, we first summarized the daily device-measured intensity values and categorized participants into quartiles (Q1–Q4), representing increasing levels of physical activity. Physical activity was further divided into daytime (7:00 AM–7:00 PM) and nighttime (7:00 PM–6:00 AM the following day) periods. Weighted multivariable logistic regression models were used to evaluate the association between physical activity and MAFLD. Two models were applied to assess the robustness of the association: Model 1 was unadjusted to show the crude relationship, while Model 2 was adjusted for potential confounders (age, sex, race, and PIR) to account for demographic and socioeconomic influences on both physical activity and MAFLD risk. Tests for linear trends across quartiles were performed in Model 3.

To examine the consistency of associations, subgroup analyses were conducted to further explore these associations, stratified by sex (male and female), age group (12–15 and 16–19 years old), PIR (≤1.3, 1.3–3.5, >3.5), and race/ethnicity (non-Hispanic White, non-Hispanic Black, Mexican American). All our data analyses were conducted using R version 4.3.3. Descriptive analyses were performed with “gtsummary” R package. A two-sided *P* <0.05 was considered statistically significant.

## Results

### Baseline characteristics of participants

After excluding participants with missing data on physical activity or MAFLD status, 5705 adolescents aged 12–19 years were included in the final analysis, comprising 3666 from the 2003–2006 cycle and 2039 from the 2011–2014 cycle. Their characteristics is shown in Table 1. The prevalence of MAFLD was 22.6 % and 20.0 % in the two cycles, respectively. The median age was 15 years in both cycles, and the sex distribution was balanced (51 % males, 49 % females). MAFLD prevalence was higher among females (58 % vs. 42 % in 2003–2006; 55 % vs. 45 % in 2011–2014). Across both cycles, MAFLD was positively associated with higher BMI, ALT, SBP, and obesity status (*P* < 0.001). A significant difference of MAFLD rate in difference physical activity quartiles was observed in 2003–2006, whereas no significant difference was found in 2011–2014.

### Physical activity associated with lower mafld risk

To examine the association between total physical activity and MAFLD, we conducted logistic regression analyses with different sets of covariates in two survey cycles, respectively. As shown in Table 2, in 2003–2006, higher total physical activity was strongly and consistently associated with lower odds of MAFLD across all models. In the fully adjusted model, Q3 and Q4 participants had 46 % and 60 % lower odds, respectively, compared with Q1. Both daytime and nighttime activity in Q4 were significantly protective, with daytime activity showing a slightly stronger association. In 2011–2014, the inverse association was weaker and not statistically significant after full adjustment.

**Fig. 2:**
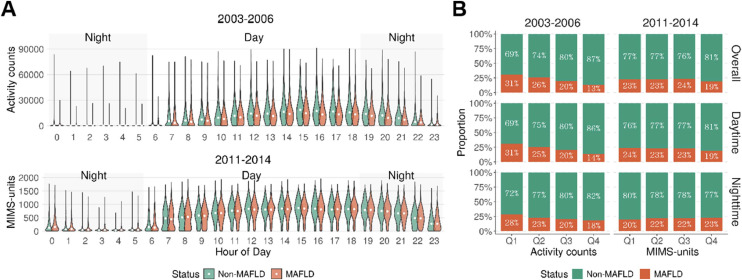
Distribution of physical activity by MAFLD status and time period.

Next, we investigated the impact of physical activity timing on MAFLD. Fig. 2A shows that physical activity was concentrated between 7:00 AM and 10:00 PM in both cycles, with non-MAFLD participants consistently exhibiting higher hourly activity levels, particularly during the day. Nighttime activity levels were generally higher in 2011–2014 compared to 2003–2006, indicating a shift in patterns over time. Fig. 2B shows that in 2003–2006, the prevalence of MAFLD decreased steadily with increasing activity levels for overall, daytime, and nighttime activity. However, for 2011–2014 cycle, the trend was less apparent.

(A) Hourly distribution of accelerometer-measured physical activity among adolescents with and without MAFLD during 2003–2006 (ActiGraph AM-7164; activity counts) and 2011–2014 (ActiGraph GT3X+; MIMS-units) survey cycle. Violin plots show activity levels by hour, with daytime (7:00–19:00) and nighttime (20:00–6:00) periods shaded. Values above the 99th percentile were excluded. (B) Proportion of adolescents with MAFLD across quartiles of overall, daytime, and nighttime physical activity levels, stratified by survey cycle.

We then divided physical activity into daytime (7:00 AM–7:00 PM) and nighttime (7:00 PM–6:00 AM) periods and conducted stratified logistic regression analyses (Table 2). In the 2003–2006 cycle, both daytime and nighttime activity in the highest quartile (Q4) were significantly associated with lower odds of MAFLD, with daytime activity showing a stronger effect. In contrast, during the 2011–2014 cycle, we found no significant association between psychical activity with MAFLD in adolescents. To ensure the robustness of our findings regarding the definition of daytime and nighttime activity, we conducted a sensitivity analysis using alternative time thresholds for day/night classification. Across all definitions, the associations between higher physical activity and lower MAFLD risk remained consistent. Detailed results are provided in Supplementary Table S1 and Supplementary Figure S1.

### Subgroup analysis of the 2003–2006 cycle

To investigate potential subgroup differences in the association between physical activity and MAFLD, we conducted logistic regression analyses stratified by sex, age, family income, and race in 2003–2006 survey cycle (Table 3).

Females exhibited a stronger inverse relationship between physical activity and MAFLD risk than males, particularly for daytime activity (Q4 vs. Q1: female OR = 0.31, male OR = 0.51). Adolescents aged 16–19 years (nighttime Q4 vs. Q1: OR = 0.52) showed a more pronounced benefit from high-intensity nighttime activity compared to those aged 12–15 years (nighttime Q4 vs. Q1: OR = 0.68). Race-stratified analyses indicated that non-Hispanic White engaging in higher-intensity activity had lower odds of MAFLD (overall Q4 vs. Q1: OR = 0.36) than that of Mexican American (overall Q4 vs. Q1: OR = 0.55) and Non-Hispanic Black (overall Q4 vs. Q1: OR = 0.53).

## Discussion

In this cross-sectional study based on NHANES data, we found that higher physical activity levels (particularly during the daytime) were significantly associated with a lower risk of MAFLD in adolescents. Although most existing evidence comes from adult populations, our findings are consistent with prior studies. For instance, a large cohort study using UK Biobank data showed that accelerometer-measured MVPA was inversely associated with incident NAFLD in adults [25]. A Korean population-based study showed that achieving the WHO-recommended volume of moderate-to-vigorous physical activity (≥150 min/week) was associated with a 14 % lower risk of NAFLD [26]. Given the consensus on the important role of circadian rhythm in physiology and disease, daytime patterns of physical activity appear relevant for health outcomes. For example, Zhang et al. reported that individuals with an early-morning physical activity pattern had higher all-cause and cardiovascular mortality compared with those active at midday, particularly when combined with irregular sleep patterns [27]. We considered that physical activity during different time periods may have an impact on the risk of MAFLD in adolescents, so we monitored the characteristics of hourly physical activity in the population and artificially divided the time into daytime and nighttime based on the circadian rhythm. The final study showed that compared to high-intensity physical activity at night, daytime reduces the risk of MAFLD in adolescents more likely. However, this association was only observed in the 2003–2006 data and not in the 2011–2014 data. A possible reason may be the physical activity monitor used in 2003–2006 was a uniaxial device worn on the right hip, whereas the monitor used in 2011–2014 was worn on the wrist and measured acceleration in three mutually perpendicular directions (x, y, and z axes). This allows it to capture movements in any direction in space, which is the most fundamental reason why the two datasets are not directly comparable. Additionally, there were differences in the lifestyle patterns of U.S. adolescents between 2003–2006 and 2011–2014. The proportion of people across all age groups in the United States using computers during leisure time increased significantly [28]. For example, the percentage of adolescents using a computer for one hour or more per day rose from 52.6 % in 2003 to 64.1 % in 2014 [28]. From 2007 to 2016, self-reported sedentary time among adolescents increased from 7.0 h per day to 8.2 h [28] and prolonged sedentary time is associated with an increased risk of obesity [29].

In our subgroup analysis, regardless of which group, the risk of MAFLD was lower during the day for the same intensity of physical activity. And we did not observe any association between nighttime physical activity and MAFLD in the data from 2011 to 2014. The reasons for the difference in the risk of illness between day and night may be because our nighttime includes physical activities and sleep during the night. A study has shown that nighttime physical activity in adolescents can cause circadian rhythm disorders [30]. Reutrakul et al. found that circadian rhythm disorders are associated with impaired cardiometabolic function as well as increased risks of obesity, diabetes, and cardiovascular diseases [31]. The mechanisms behind these associations may still need more research to explore. What can be clearly stated is that physical activity during the day can significantly reduce the risk of MAFLD in adolescents.

Our study indicates that white women who engage in high-intensity physical activity have the lowest risk of developing MAFLD, which may be attributed to the role of estrogen in enabling more efficient utilization of fat as fuel during high-intensity exercise [32,33]. Additionally, genetic differences in muscle fiber composition and muscular metabolic efficiency may exist among different racial groups. These differences could influence individual metabolic responses to high-intensity exercise, leading to varying effectiveness in reducing MAFLD risk [34]. Furthermore, white populations may enjoy more favorable socioeconomic status, providing them with greater capacity and opportunities to maintain a healthy lifestyle that includes high-intensity exercise over the long term [35].

However, current research has some limitations. Firstly, due to the cross-sectional design used in this study, the observed connections do not necessarily indicate a causal relationship. Additionally, factors such as weather conditions or seasonal variations may lead to differences in adolescents' physical activity levels, which we were unable to capture in our data. Secondly, we used indirect methods (HSI assessment tools) rather than imaging studies or pathological evaluations to determine MAFLD. While HSI has been validated as a non-invasive screening, it remains less precise than imaging or biopsy. Thirdly, there is no direct evidence that MIMS units can be converted into standardized measures of moderate-to-vigorous physical activity, and results may vary when using different accelerometer devices. Finally, the physical activity monitor used in 2003–2006 was a uniaxial accelerometer that measured only vertical acceleration. As a result, any activities not primarily generating motion in the vertical direction were likely underestimated. However, it accurately captured walking, running, and daily activities for most people, the conclusions of this study remain valid despite this limitation.

## Conclusion

In this large national cross-sectional study, we demonstrated a potential protective association between physical activity measured by accelerometer and adolescent MAFLD, with stronger physical activity leading to a lower risk of MAFLD in adolescents. The association was more evident for daytime physical activity.

**Table 1:** Characteristics of participants in the NHANES 2003–2006 and 2013–2014 cycles, stratified by physical activity (Q1-Q4).

Characteristics	2003–2006 (*N* = 3666)				2013–2014 (*N* = 2039)			
	Overall (*N* = 3666)	Non-MAFLD (*N* = 2837)	MAFLD (*N* = 829)	*P*	Overall (*N* = 2039)	Non-MAFLD (*N* = 1591)	MAFLD (*N* = 448)	*P*
Age, years	15.5 (2.3)	15.3 (2.3)	16.2 (2.2)	< 0.001	15.4 (2.3)	15.2 (2.2)	16.0 (2.2)	< 0.001
BMI, kg/m	24.1 (6.1)	21.5 (3.1)	33.1 (5.7)	< 0.001	24.1 (6.3)	21.5 (3.3)	33.3 (5.7)	< 0.001
ALT, U/L	20.0 (16.0)	17.7 (11.8)	27.9 (23.9)	< 0.001	19.1 (13.1)	17.0 (8.5)	26.6 (21.2)	< 0.001
AST, U/L	24.5 (11.8)	24.4 (11.8)	24.8 (11.8)	0.027	23.6 (11.1)	23.4 (11.2)	24.1 (10.5)	0.200
SBP, mmHg	110.3 (10.4)	109.0 (10.0)	114.9 (10.4)	< 0.001	109.0 (9.9)	107.8 (9.5)	113.0 (10.2)	< 0.001
DBP, mmHg	59.4 (11.6)	59.4 (11.4)	59.6 (12.2)	0.500	58.3 (13.2)	57.7 (13.0)	60.3 (13.5)	< 0.001
Sex				< 0.001				0.006
Male	1875 (51.1)	1529 (53.8)	346 (42.1)		1036 (50.8)	834 (52.4)	202 (45.1)	
Female	1791 (48.9)	1315 (46.2)	476 (57.9)		1003 (49.2)	757 (47.6)	246 (54.9)	
Race				0.027				0.029
Mexican American	1181 (32.2)	892 (31.4)	289 (35.2)		429 (21.0)	320 (20.1)	109 (24.3)	
Non-Hispanic White	963 (26.3)	777 (27.3)	186 (22.6)		499 (24.5)	392 (24.6)	107 (23.9)	
Non-Hispanic Black	1260 (34.4)	967 (34.0)	293 (35.6)		533 (26.1)	406 (25.5)	127 (28.3)	
Other	262 (7.1)	208 (7.3)	54 (6.6)		578 (28.3)	473 (29.7)	105 (23.4)	
Family income				< 0.001				< 0.001
Low	1461 (39.9)	1098 (38.6)	363 (44.2)		846 (41.5)	631 (39.7)	215 (48.0)	
Medium	1290 (35.2)	997 (35.1)	293 (35.6)		644 (31.6)	500 (31.4)	144 (32.1)	
High	915 (25.0)	749 (26.3)	166 (20.2)		549 (26.9)	460 (28.9)	89 (19.9)	
Diabetes				0.021				
No	3652 (99.6)	2837 (99.8)	815 (99.1)		2029 (99.5)	1586 (99.7)	443 (98.9)	0.047
Yes	14 (0.4)	7 (0.2)	7 (0.9)		10 (0.5)	5 (0.3)	5 (1.1)	
Obesity				< 0.001				< 0.001
No	1632 (44.2)	1631 (57.0)	1 (0.01)		933 (45.2)	933 (58.1)	0 (0.0)	
Yes	2034 (55.8)	1213 (43.0)	821 (99.9)		1106 (54.8)	658 (41.9)	448 (100.0)	
Physical activity				< 0.001				0.400
Q1	917 (25.0)	635 (22.3)	282 (34.3)		510 (25.0)	395 (24.8)	115 (25.7)	
Q2	916 (25.0)	679 (23.9)	237 (28.8)		509 (25.0)	394 (24.8)	115 (25.7)	
Q3	916 (25.0)	735 (25.8)	181 (22.0)		510 (25.0)	390 (24.5)	120 (26.8)	
Q4	917 (25.0)	795 (28.0)	122 (14.8)		510 (25.0)	412 (25.9)	98 (21.9)	

Note: Categorical variables are presented as N ( %), and continuous variables are presented as mean (SD). P values indicate differences between MAFLD and non-MAFLD groups.

**Table 2:** Association between physical activity and MAFLD.

Physical activity	2003–2006						2011–2014					
	Overall		Day-time (7 AM to 7 PM)		Night-time (8 PM to 6 AM)		Overall		Day-time (7 AM to 7 PM)		Night-time (8 PM to 6 AM)	
	OR (95 %CI)	*P*	OR (95 %CI)	*P*	OR (95 %CI)	*P*	OR (95 %CI)	*P*	OR (95 %CI)	*P*	OR (95 %CI)	*P*
Model 1												
Q1	Ref		Ref		Ref		Ref		Ref		Ref	
Q2	0.95 (0.74, 1.23)	0.707	1.04 (0.77, 1.40)	0.817	0.67 (0.52, 0.87)	0.005	0.89 (0.57, 1.39)	0.604	0.70 (0.46, 1.06)	0.099	1.12 (0.70, 1.80)	0.637
Q3	0.53 (0.38, 0.75)	0.001	0.62 (0.46, 0.82)	0.003	0.64 (0.46, 0.88)	0.010	0.80 (0.49, 1.31)	0.382	0.75 (0.49, 1.13)	0.179	1.15 (0.75, 1.77)	0.520
Q4	0.34 (0.26, 0.45)	< 0.001	0.36 (0.26, 0.48)	< 0.001	0.58 (0.46, 0.73)	< 0.001	0.60 (0.39, 0.95)	0.037	0.53 (0.33, 0.85)	0.013	1.18 (0.76, 1.84)	0.465
Trend	0.70 (0.64, 0.76)	< 0.001	0.72 (0.66, 0.78)	< 0.001	0.84 (0.77, 0.90)	< 0.001	0.85 (0.74,0.98)	0.038	0.83 (0.72,0.96)	0.018	1.05 (0.92, 1.21)	0.466
Model2												
Q1	Ref		Ref		Ref		Ref		Ref		Ref	
Q2	1.01 (0.78, 1.30)	0.951	1.08 (0.80, 1.46)	0.599	0.67 (0.52, 0.86)	< 0.001	0.90 (0.57, 1.42)	0.654	0.71 (0.47, 1.08)	0.120	1.11 (0.68, 1.82)	0.671
Q3	0.59 (0.42, 0.83)	< 0.001	0.69 (0.51, 0.92)	0.02	0.66 (0.47, 0.91)	0.018	0.89 (0.54, 1.48)	0.665	0.86 (0.56, 1.33)	0.499	1.10 (0.72, 1.70)	0.664
Q4	0.41 (0.31, 0.55)	< 0.001	0.44 (0.31, 0.61)	< 0.001	0.58 (0.45, 0.75)	< 0.001	0.68 (0.43, 1.08)	0.115	0.66 (0.41, 1.07)	0.106	1.02 (0.64, 1.61)	0.937
Trend	0.74 (0.68, 0.82)	< 0.001	0.77 (0.70, 0.85)	< 0.001	0.84 (0.77, 0.92)	0.000	0.89 (0.77, 1.04)	0.146	0.90 (0.77, 1.04)	0.169	1.01 (0.87, 1.16)	0.941
Model3												
Q1	Ref		Ref		Ref		Ref		Ref		Ref	
Q2	0.98 (0.76, 1.26)	0.872	1.07 (0.81, 1.42)	0.647	0.67 (0.52, 0.88)	0.009	0.86 (0.54, 1.37)	0.521	0.66 (0.43, 1.02)	0.073	1.17 (0.69, 1.97)	0.560
Q3	0.54 (0.39, 0.74)	< 0.001	0.62 (0.48, 0.81)	0.002	0.62 (0.45, 0.87)	0.012	0.87 (0.51, 1.47)	0.603	0.82 (0.52, 1.28)	0.387	1.16 (0.76, 1.76)	0.503
Q4	0.40 (0.30, 0.53)	< 0.001	0.42 (0.30, 0.60)	< 0.001	0.57 (0.44, 0.75)	< 0.001	0.65 (0.41, 1.03)	0.080	0.63 (0.38, 1.05)	0.089	0.97 (0.63, 1.50)	0.895
Trend	0.73 (0.67, 0.80)	< 0.001	0.75 (0.69, 0.82)	< 0.001	0.83 (0.76, 0.91)	< 0.001	0.88 (0.76, 1.03)	0.117	0.89 (0.76, 1.04)	0.147	0.99 (0.87, 1.14)	0.914

Note: Model 1 shows unadjusted estimates. Model 2 adjusted for age and sex. Model 3 further adjusted for race and family income.

**Table 3:** Subgroup analysis of the association between physical activity and MAFLD (03–06 cycle).

Physical activity	Sex		Age		PIR			Race		
	Male	Female	12–15 years	16–19 years	Low	Medium	High	Mexican American	Non-Hispanic White	Non-Hispanic Black
Overall										
Q1	Ref	Ref	Ref	Ref	Ref	Ref	Ref	Ref	Ref	Ref
Q2	0.99 (0.58, 1.69)	0.97 (0.71, 1.31)	1.35 (0.90, 2.03)	0.80 (0.53, 1.22)	0.65 (0.45, 0.94)	1.03 (0.61, 1.71)	1.44 (0.87, 2.37)	0.85 (0.58, 1.25)	1.05 (0.70, 1.57)	0.70 (0.48, 1.01)
Q3	0.54 (0.32, 0.92)	0.55 (0.39, 0.76)	0.67 (0.44, 1.01)	0.47 (0.30, 0.75)	0.50 (0.32, 0.78)	0.60 (0.33, 1.10)	0.52 (0.26, 1.01)	0.70 (0.47, 1.04)	0.54 (0.33, 0.88)	0.59 (0.41, 0.87)
Q4	0.46 (0.30, 0.69)	0.32 (0.19, 0.53)	0.38 (0.25, 0.58)	0.48 (0.31, 0.73)	0.37 (0.21, 0.65)	0.45 (0.28, 0.74)	0.37 (0.17, 0.81)	0.55 (0.35, 0.85)	0.36 (0.21, 0.64)	0.53 (0.35, 0.80)
Day-time										
Q1	Ref	Ref	Ref	Ref	Ref	Ref	Ref	Ref	Ref	Ref
Q2	1.12 (0.74, 1.68)	1.06 (0.71, 1.58)	1.69 (1.13, 2.55)	0.81 (0.56, 1.16)	0.56 (0.41, 0.77)	1.36 (0.81, 2.29)	1.57 (0.94, 2.62)	0.71 (0.48, 1.04)	1.15 (0.77, 1.73)	0.68 (0.47, 0.98)
Q3	0.66 (0.43, 1.01)	0.59 (0.42, 0.83)	0.76 (0.54, 1.06)	0.56 (0.37, 0.84)	0.59 (0.38, 0.90)	0.59 (0.35, 0.97)	0.70 (0.40, 1.23)	0.75 (0.50, 1.11)	0.62 (0.39, 0.99)	0.59 (0.41, 0.87)
Q4	0.51 (0.34, 0.75)	0.31 (0.16, 0.63)	0.40 (0.23, 0.69)	0.53 (0.33, 0.85)	0.33 (0.17, 0.63)	0.55 (0.32, 0.93)	0.40 (0.20, 0.81)	0.56 (0.36, 0.87)	0.36 (0.20, 0.64)	0.53 (0.34, 0.82)
Night-time										
Q1	Ref	Ref	Ref	Ref	Ref	Ref	Ref	Ref	Ref	Ref
Q2	0.83 (0.54, 1.27)	0.59 (0.37, 0.94)	0.79 (0.49, 1.25)	0.62 (0.45, 0.84)	0.78 (0.53, 1.16)	0.83 (0.49, 1.40)	0.47 (0.26, 0.86)	0.93 (0.65, 1.33)	0.75 (0.49, 1.16)	0.70 (0.46, 1.06)
Q3	0.66 (0.36, 1.22)	0.60 (0.44, 0.81)	0.88 (0.53, 1.44)	0.47 (0.31, 0.71)	0.76 (0.47, 1.24)	0.69 (0.38, 1.27)	0.39 (0.19, 0.80)	0.76 (0.51, 1.12)	0.69 (0.44, 1.09)	0.57 (0.38, 0.85)
Q4	0.63 (0.38, 1.04)	0.54 (0.36, 0.81)	0.68 (0.43, 1.06)	0.52 (0.36, 0.74)	0.53 (0.34, 0.84)	0.65 (0.40, 1.06)	0.55 (0.27, 1.13)	0.64 (0.41, 0.98)	0.60 (0.37, 0.97)	0.62 (0.42, 0.91)

Note: covariates included age, sex, race, and family income, except for the variable used for stratification in subgroup analysis.

## Data availability

The data used in this study are publicly available at the NHANES database. Analysis code is available at: https://zhanghaoyang.net/project/mafld_pa/.

## CRediT authorship contribution statement

**Ruli Wang:** Writing – original draft, Formal analysis, Data curation, Conceptualization. **Congwei You:** Formal analysis, Data curation. **Zijuan Dong:** Formal analysis, Data curation. **Qingqing Zheng:** Writing – review & editing. **Xiaowei Zheng:** Methodology. **Liangbin Zhou:** Writing – review & editing. **Xiaolei Wang:** Supervision. **Le Zhang:** Supervision, Funding acquisition. **Haoyang Zhang:** Writing – original draft, Supervision, Data curation.

## Declaration of competing interest

We wish to confirm that there are no known conflicts of interest associated with this publication and there has been no significant financial support for this work that could have influenced its outcome

## Appendix Supplementary materials

Image, image 1

Image, application 2
