# Supplementary figures and images for: Wearable device–measured physical activity and risk of MAFLD in adolescents
Source: Am J Prev Cardiol. 2025 Nov 3;24:101345. doi: 10.1016/j.ajpc.2025.101345 (PMC12746288; doi:10.1016/j.ajpc.2025.101345)

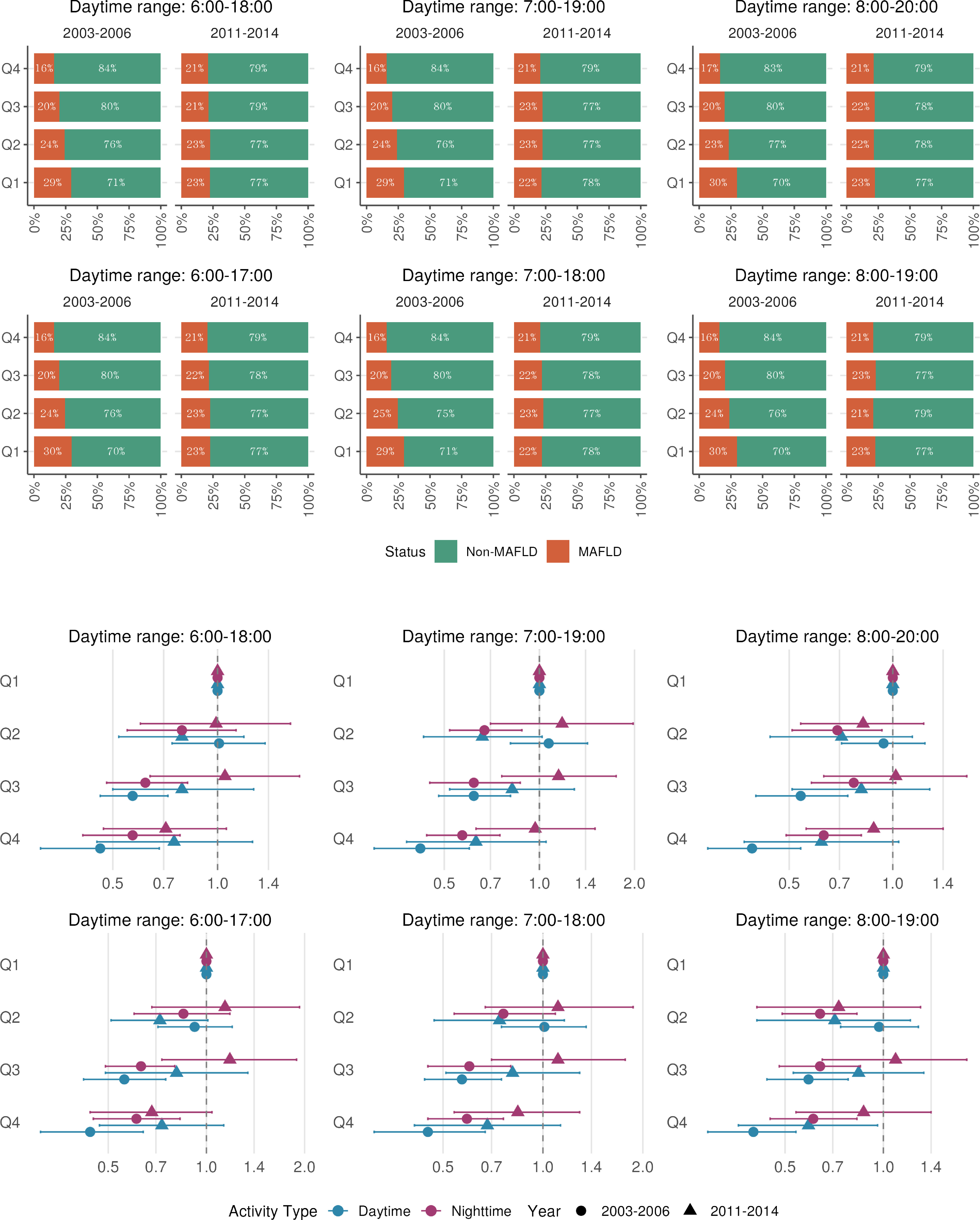

Supplement: Supplementary file 1 [file mmc1.jpg]
